# Effects of Different Drying Methods on Drying Characteristics and Quality of *Glycyrrhiza uralensis* (Licorice)

**DOI:** 10.3390/foods12081652

**Published:** 2023-04-15

**Authors:** Lichun Zhu, Mengqing Li, Wenxin Yang, Junyi Zhang, Xuhai Yang, Qian Zhang, Huting Wang

**Affiliations:** 1 College of Mechanical and Electrical Engineering, Shihezi University, Shihezi 832003, China; zhulichun0204@126.com (L.Z.);; 2 Engineering Research Center for Production Mechanization of Oasis Special Economic Crop, Ministry of Education, Shihezi 832003, China; 3 Xinjiang Production and Construction Corps Key Laboratory of Modern Agricultural Machinery, Shihezi 832003, China

**Keywords:** *Glycyrrhiza uralensis* (Licorice), drying methods, drying characteristics, total flavonoid content (TFC), total phenolic content (TPC), active ingredient

## Abstract

Large amounts of waste result from licorice mold rot; moreover, prompt drying directly influences product quality and value. This study compared various glycyrrhiza drying methods (Hot air drying (HAD), infrared combined hot air drying (IR-HAD), vacuum freeze drying (VFD), microwave vacuum drying (MVD), and vacuum pulsation drying (VPD)) that are used in the processing of traditional Chinese medicine. To investigate the effects of various drying methods on the drying characteristics and internal quality of licorice slices, their color, browning, total phenol, total flavonoid, and active components (liquiritin and glycyrrhizic acid) were chosen as qualitative and quantitative evaluation indices. Our results revealed that VFD had the longest drying time, but it could effectively maintain the contents of total phenol, total flavonoid, and liquiritin and glycyrrhizic acid. The results also showed that VFD samples had the best color and the lowest degree of browning, followed by HAD, IR-HAD, and VPD. We think that VFD is the best approach to ensure that licorice is dry.

## 1. Introduction

The COVID-19 epidemic and its recurrence have had a tremendous influence on the world and the virus’s constant evolution has made the discovery of medications to prevent outbreaks extremely difficult. Since *Glycyrrhiza uralensis* (Licorice) is a traditional Chinese medicine; it is frequently used to treat a variety of illnesses, including cancer, cardiovascular and cerebrovascular diseases, and liver inflammation. Its primary ingredient, glycyrrhizic acid, was first mentioned as an adjuvant in the treatment of SARS as early as 2005 [1]. A significant number of studies conducted in recent years have demonstrated that glycyrrhizic acid can decrease intracellular oxidative stress, lower the expression of the high mobility group protein B1 (HMGB1), and inhibit the expression and activity of nuclear factor kappa B (NF-κB) [2], which is crucial for the prevention and treatment of inflammation. Licorice is unquestionably the most effective treatment for some people with visceral weakness, weakness, and other symptoms. In addition, it can be used to heal ulcers, sore throats, and other illnesses. It may be applied physically, taken orally, combined with other medications, or used alone. In addition, licorice, can be used in conjunction with other medications to enhance therapy [3], can be used to alleviate pain in many digestive system organs. Acute pain is obviously relieved by licorice. It can also have unusual anti-inflammatory and anti-allergy properties, and it can successfully reduce respiratory system irritation.

China has large stocks of licorice, which it consumes at a rate of 50,000–60,000 tons annually. However, licorice is susceptible to moisture in the air for a long time after harvest and to mycotoxins produced by various fungi, which can cause degradation and mildew [4]. This not only compromises the quality and safety of traditional Chinese medicine and its processed products but also poses a serious threat to people’s health and quality of life. Productivity of certain semi-finished goods, such as licorice slices, is insufficient due to a lack of theoretical study, specifically on the practical application of the drying process of raw materials. Knowledge gaps regarding the initial drying process are important issues that need to be resolved. There are strict criteria for the licorice’s drying quality when it comes to storage, processing, and shipping of dried goods. Currently, the primary method for drying licorice is still conventional natural drying, in which the root is dug up from the ground and spread out on a drying field [5]. This approach requires large amounts of space and time to dry, and wastes natural resources. The loss of active ingredients in licorice dried under natural settings is significant, the hygienic environment cannot be assured, and it is easily contaminated by insects and flies when rainy. In contrast, the hot-air drying method is widely used in licorice processing [6] because it is not affected by weather, climate, or other natural factors, and the corresponding drying temperature can be controlled based on the efficacy characteristics of the licorice, but the utilization rate of thermal efficiency is low and the heating time is long.

The use of new drying technology in the drying of Chinese herbal medicine has grown rapidly in recent years. Initial processing at the point of origin is a critical link in the industrial chain of traditional Chinese medicine [7]. However, there is little research on licorice drying. Approximately 70% of the commonly used Chinese medicinal materials in China must be processed at the point of origin. It is clear that drying is both universal and important in the production of traditional Chinese medicine. Existing drying technologies primarily include infrared combined hot air drying, vacuum freeze drying, microwave vacuum drying, vacuum pulsating drying, and others. Each has their respective advantages, such as infrared combined hot air drying, which dries materials through infrared radiation has high efficiency, and is energy saving [8]; Vacuum freeze-drying retains nutrients to a great extent and the structure is not easily deformed, thus, has quality advantages in fruit and vegetable processing [9]. Microwave vacuum drying greatly improves the drying rate through microwave penetration heating [10]. Vacuum pulsating drying can protect the microstructure and color of materials [11]. Chinese herbal medicine products are evaluated primarily by their internal quality and external characteristics. Measured efficacy is used to assess internal quality, and external characteristics including the weight, shape, color, and texture of Chinese herbal medicine are used to assess external quality. These techniques have been widely used in paeoniflora [12], Moutan bark [13], panax notoginseng [14], burdock [15], aconite [16], Danshen [17], pueraria [18] and other medicinal materials. Dried traditional Chinese medicine products obtained by high-tech drying methods are of high quality, are dried rapidly, and have little loss of active ingredients by avoiding the loss of heat-sensitive components. At the same time, oxidative denaturation of the components is reduced. Furthermore, the benefits of environmental protection and sterilization are significant.

In this study, licorice root was cleaned as it was gathered, then sliced, in accordance with extensive research on the basic processing techniques used in traditional Chinese medicine. Hot air drying (HAD), infrared combined hot air drying (IR-HAD), vacuum freeze drying (VFD), microwave vacuum drying (MVD), and vacuum pulsation drying (VPD) were all used to dry the licorice. Qualitative and quantitative evaluation indices were chosen: drying properties of licorice tablets (moisture content, drying rate, effective diffusion coefficient of water), color, browning, total phenol, total flavonoid, and active ingredient (Liquiritin and glycyrrhizic acid) [19,20]. In order to improve the initial drying processing methods of licorice in the producing area, the impacts of various drying methods on the drying characteristics and internal quality of licorice slices were investigated. In order to guarantee the quality and clinical safety of licorice, the goal of this study is to establish a theoretical foundation for standardizing the initial processing technology of licorice tablets.

## 2. Materials and Methods

**Figure 1:**
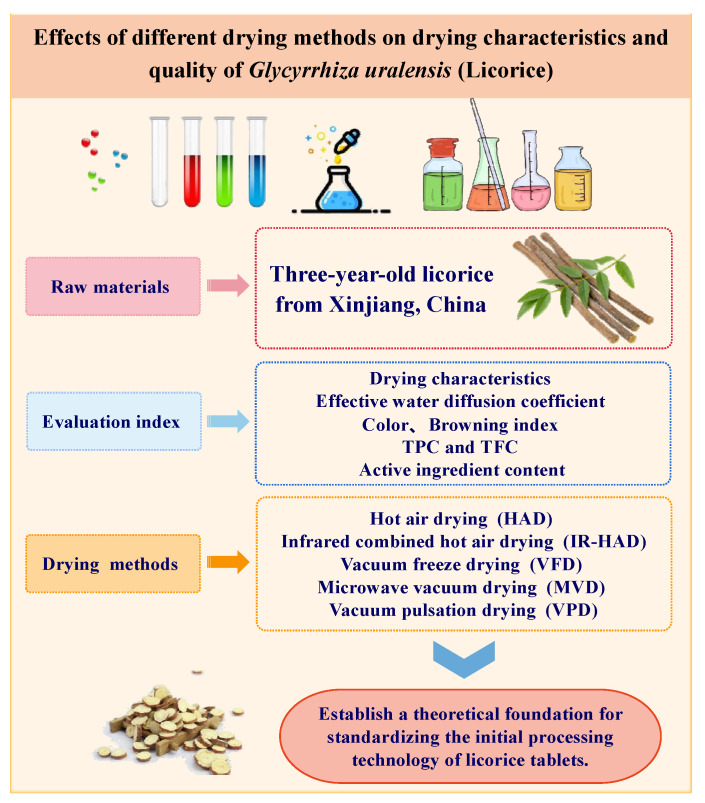
Schematic overview of the experimental procedure.

The overall experimental setup process is shown in Figure 1.

### 2.1. Materials

Fresh licorice was obtained from Ural types that had been planted in the 49 regiment of Tumushuke City, the third division in the south of Tianshan, Xinjiang, China. All raw ingredients of three-year-old medicinal materials were extracted from underground roots at a cost of 23 RMB/kg, and they were kept at 4℃ in an air-conditioned storage. Before the experiment licorice were cleansed (as they are not washable) as follows: mud and sand on root strips were cleaned, and fibrous roots and other inactive components of licorice roots were eliminated. Pre-selected and thinly-sliced licorice root strips with a similar diameter (15 ± 3 mm) were dried in order to assure the accuracy of the findings and minimize error.

### 2.2. Instruments and Equipment

Licorice slices were dried using five different techniques in this experiment. The drying equipment used included: hot-air drying oven (DHG-9070 A, power 1550 W, voltage 220 V, temperature adjustment range RT +1 0-250C, Shanghai Yiheng Science and Technology Co., Ltd., Shanghai, China); infrared combined hot-air drying oven (Laboratory of agricultural products processing technology and equipment, School of Mechanical and Electrical Engineering, Shihezi University); vacuum freeze-drying oven (sequence 22899, power 0.75 kVA, voltage 230 V, CHRIST Company, Osterode, Germany); microwave vacuum drying oven (RWBZ-08 S, microwave power 800 W, voltage 220 V, Nanjing Su Enrui drying equipment Co., Ltd., Nanjing, China); and vacuum pulsating drying oven (VD-6090D, heating power consumption 7 kW, voltage 220 V, Suzhou Yinuohuake Industrial equipment Co., Ltd., Suzhou, China). In order to obtain a stable functioning state for testing, machines were preheated prior to all drying tests; drying equipment were operated normally under the appropriate parameters for 30 min beforehand.

The composition determination equipment used included: ACQUITY UPLC H-class ultra high performance liquid chromatograph, XEVO TQS triple quadrupole tandem mass spectrometer, equipped with MassLynx software (Waters Company, Milford, MA, USA); Centrifuge 5427R high speed freezing centrifuge (Eppendorf Company, Hamburg, Germany); L6-180 ultrasonic cleaning machine (Shanghai Haozhuang instrument Co., Ltd., Shanghai, China); rotary evaporation instrument (Heidolph Company, Schwabach, Germany); Milli-Q ^®^IQ 7000 pure water machine (Milibo Company, Shanghai, China). Ms1602Ts 1/100000 Electronic Analytical balance (Mettler-Toledo Instrument Co., Ltd., Shanghai, China); and FW high-speed universal grinder (Beijing Yongming Medical instrument Factory, Beijing, China).

Other equipment used included: electronic weighing balance (precision 0.01JA1003, Shanghai Precision Scientific Instrument Co., Ltd., Shanghai, China); color difference meter (model CR-400; Japan Minolta); and others.

### 2.3. Experimental Procedure

When choosing fresh licorice for drying, we looked for uniform thickness, a full root shape, a full feel, and absence of damage or decay. Licorice that had been sorted was cleaned with a brush, cross-cut into slices 3–4 mm thick, weighed with a scale that has a precision of 0.01 g, and lain flat on the material plate.

1. Relevant parameters of the tests were set as follows: HAD: drying medium temperature, 60 °C; air speed, 2.2 m/s; IR-HAD: drying medium temperature, 60 °C; IR power, 675 W; air speed, 2.5 m/s; VPD: drying medium temperature, 60 °C; atmospheric pressure; holding time, 15 min; vacuum holding time, 5 min; VFD: cold-trap temperature, −53 °C; drying chamber pressure, 12 Pa; MVD: drying medium temperature, 60 °C; microwave power, 450 W. The start button was opened and the drying box preheated until the set temperature was reached in the drying room.
2. Licorice slices with a mass of approximately 50 g were weighed using an electronic balance, and the single layer was lain flat and placed on the loading plate of the already stable drying box to begin drying.
3. The drying method was selected as the experimental factor.
4. After the start of the experiment, the samples were weighed and recorded with an electronic balance at regular intervals (samples were measured at least 6 times during the drying test, and each measurement process took less than 30 s).
5. According to the measured material quality (the prescribed quality standard is 50 g), drying could be stopped until the moisture content was less than 10 percent, the dried product was cooled to room temperature and bagged according to number and sealed in a fresh-keeping bag. Then the drying method was changed for the next set of tests (repeated 3 times in each group).
6. After all the tests were completed, the machine was turned off and the test-bed was cleaned.

### 2.4. Analysis of Drying Characteristics

#### 2.4.1. Initial Moisture Content

The initial moisture content of licorice was measured by direct drying in a 105 °C oven [21]. The test licorice was cut into slices, then 30 g samples were weighed and placed in a 105 °C oven for heating and drying.

The initial moisture content of licorice was calculated according to Equation (1):

(1) $$W_{0}=\frac{M_{0}-M_{\mathrm{ad}}}{M_{0}}$$

where *W*_0_ is initial moisture content of licorice (%), *M*_0_ is the licorice slice initial quality before drying (kg), and *M_ad_* is the licorice slice absolute dry quality (kg).

#### 2.4.2. Safe Moisture Content

Safe moisture content is required for long-term storage and is achieved by reducing water content. Whether a material can be stored for a long time or not depends on its moisture content when stored and the temperature when stored. Therefore, after drying, the moisture content of the material should reach a certain degree before long-term storage. According to the standard of Chinese Pharmacopoeia (2020 edition) and drying test experience [21], the safe moisture content of licorice slices is not higher than 10%.

#### 2.4.3. Moisture Ratio (MR)

The *MR* of licorice was calculated according to Equation (2), as reported previously [22]:

(2) $$MR=\frac{M_{t}-M_{e}}{M_{0}-M_{e}}$$

where *M_t_* is the dry basis moisture content of licorice (kg of water/kg of dry matter) at time *t*, *M_0_* at t = 0 is the initial dry basis moisture content of licorice (kg of water/kg of dry matter), and *M_e_* is the equilibrium moisture content (kg of water/kg of dry matter).

#### 2.4.4. Effective Moisture Diffusivity (*D*_eff_)

The licorice drying process is divided into several stages, among which, the main one is the slow drying stage. In this stage, the drying characteristics of the material can be calculated by Fick’s second law of effective diffusion coefficient of water, which is applied to materials with small shape variables. Through experiments, we found that licorice showed a constant deceleration stage throughout the process, so this method was suitable for this study. *D*_eff_ was measured according to Equation (3), as reported previously [8]:

(3) $$D_{\mathrm{eff}}=-\frac{L^{2}}{\pi^{2}}k$$

where *L* is the thickness of the licorice slice (mm) and *k* is the slope of the lnMR-t graph.

### 2.5. Color Attributes

Color characterization [23] is one of the most important food quality evaluation methods. Here, we used the CIELAB color table system (also known as L* a* b* color table system), combined with a slight modification of the determination method by Xu Ye et al. [24]. The measuring instrument used is a color difference meter (model CR-400; Japan Minolta). Briefly, random pieces of dried licorice powder were taken from the sample group for powder dressing. Then, L*, a*, and b* color values of dried licorice powder were measured with a color difference meter and the total color difference value (Δ*E*) was calculated. Three parallel samples were processed each time and the average value was taken. Fresh samples were used as controls. The smaller the Δ*E* value, the closer the color of the sample was to that of the original sample, and the better the color quality.

### 2.6. Browning Index

The determination method for the degree of browning was based on the determination method of Tan Shudan et al. [25], but slightly modified. Its steps were as follows: take 1 g of dried licorice sample, add 2 mL distilled water five times, grind the homogenate, centrifuge it at 10,000 r/min for 30 min, and use a Shimazu UV-1900i spectrophotometer to measure its absorbance at 420 nm wavelength.

### 2.7. Total Phenol and Total Flavonoid

The total phenol content (TPC) and total flavonoid content (TFC) were determined by ultraviolet spectrophotometry.

TPC was measured [26] using the Folin-Ciocalteu method (FC method, also known as gallic acid equivalence, or GAE method). With gallic acid as the standard substance, the absorbance value of the standard solution was determined at 760 nm. The TPC of the sample was expressed as TAE (the appropriate value of gallic acid). TFC was measured using the NaNO_2_-AlCl_3_-NaOH method [27]. Rutin solution with concentrations of 60, 80, 100, 120, 160 and 200 μg/m L were prepared. The absorption value of the standard solution was determined at 510 nm, and the standard curve of rutin was drawn. The results of the determination of TFC in the sample solution were expressed as rutin equivalent (mg/g (dry basis)).

### 2.8. Major Active Ingredients

The HPLC method [28] was used to determine the content of liquiritin and glycyrrhizic acid in licorice samples. The liquiritin control product (lot number J1016AS, containing > 98%) and glycyrrhizic acid control product (lot number D0708AS, containing > 98%) were provided by Dalian Meilen Biotechnology Co., Ltd. Methanol, Dalian, China. Acetonitrile and phosphoric acid were chromatographically pure. All other reagents were analytically pure and the water was ultra-pure.

Preparation of mixed standard solution: Standard liquiritin and glycyrrhizic acid were accurately weighed and dissolved into 1.0 mg/mL reserve solution with methanol. Appropriate amount of the above reserve liquid was accurately measured and diluted with methanol into 1, 10, 50, 100, 250, 500, 750, 1000 ng/mL mixed standard liquids.

Chromatographic conditions: The determination was performed on ACQIUTY UPLC BEH C18 (1.7 μm, 2.1 mm × 50 mm) column with temperature at 30 °C, flow rate at 0.3 mL/min, and sample size at 1.0 μL. The final optimized mobile phase: 0.1% formic acid-water (A), acetonitrile (B) solution, gradient elution: 0–2.5 min, 20–98% B; 2.5–3.5 min, 98% B; 3.5–3.6 min, 98–20% B; 3.6–5.0 min, 20% B.

Mass spectrum conditions: Electrospray ion source; ion source temperature, 150 °C; capillary voltage, 2.0 KV; ion source compensation voltage, 50 V; desolvent temperature, 450 °C; desolvent gas flow rate, 800 L/Hr; cone hole gas flow rate, 150 L/Hr; atomized gas pressure, 7.0 Bar; and multi-reaction monitoring mode (MRM). The linear equation of each component is shown in Table 1.

## 3. Results and Discussion

### 3.1. Drying Characteristics

**Figure 2:**
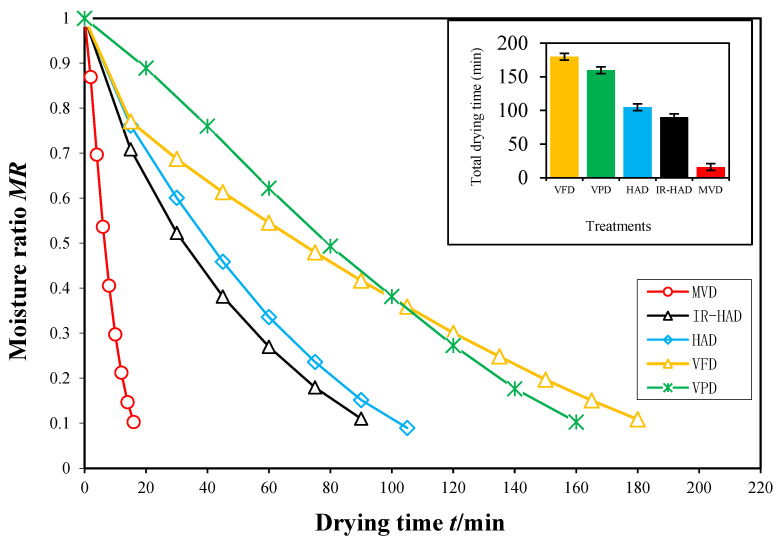
Drying kinetics curve of licorice at different drying methods.

As shown in Figure 2, among the five different drying methods, the time taken by licorice samples to reach a safe moisture content was highly variable. Microwave vacuum drying took the shortest time (16 min). If only the drying cycle time is considered, this is undoubtedly the best method. The time for VFD was the longest (180 min), while VPD was slightly shorter (160 min). Times for HAD and IR-HAD were similar (105 and 90 min, respectively). In all five different drying methods, the drying curve showed the same logarithmic trend, indicating that licorice dehydrated rapidly in the initial stage and slowly at the later drying stage. This is consistent with the results from Liu He et al. [29] on the drying characteristics of other materials. Among the drying curves, the MVD curve trend was significantly steeper than that of other drying methods. This may be because MVD increases the vapor pressure difference between the materials and the water vapor in the drying chamber, improves the activity of water molecules and weakens the binding force between them, hence, destroying the internal chemical bond. Thus, it transforms the bound water in the cell into free water with better fluidity, reducing the diffusion boundary inside the materials and enhancing mass and heat transfer efficiency.

### 3.2. Effective Water Diffusion Coefficient

The effective diffusion coefficient of water was used to represent the average rate of water migration in the drying process of licorice under different drying methods [30]. When licorice absorbs heat from the drying medium, heat is transferred from the outside into the inside, hence, water is transferred from the inside to the outside. When the critical moisture content is reached, the drying process ends. Here, we linearly fitted the ln MR of licorice and drying time t using different drying methods. As seen in Table 2, the highest water diffusion coefficient for microwave vacuum drying was 139.06; the drying rate for MVD was too fast. As a result, the diameter and fresh sample size of licorice slices changed greatly after drying, leading to serious shrinkage and low permeability inside the slices. The water diffusion coefficients for HAD, IR-HAD, and VPD were 23.86, 21.50, and 17.23, respectively. At −53 °C, the lowest moisture drying coefficient for VFD was 11.52. Since the temperature was low, the energy of the water molecules decreased and water evaporation became slow, which weakened its diffusion ability, resulting in the lowest effective water diffusion coefficient. With the exception of VPD, which had an R^2^ < 0.9, the R^2^ values of all drying methods were > 0.9. The lower R^2^ for VPD may be due to the fluctuations caused by the alternating cycle of atmospheric pressure and vacuum in the drying process [31].

### 3.3. Effect of Drying Methods on the Color of Licorice

During drying, samples are affected by enzymatic browning and non-enzymatic browning, leading to color changes [32]. The brightness, or L* value, of dried licorice reflects its degree of browning. Table 3 shows that the L* value of the VFD samples was the highest. However, there was no statistically significant difference between the IR-HAD and VPD L* values, there was little difference between the IR-HAD and VPD L* values, and the HAD L* value was the lowest and was significantly different from those of the other four groups. This demonstrates that browning can occur throughout the drying process of licorice and that heat and oxidation are key factors in promoting browning [33]. The higher the amount of oxygen present and the more severe the browning, the longer the heating period is during the drying process. Yellow was the characteristic color of licorice and there were significant differences in b* value (yellowness value) among different drying samples. The b* value of dried licorice from VFD was the highest and that from HAD was the lowest, indicating that the original yellowness of licorice could be well-maintained by VFD. The study of Gao Qingqing et al. [34] on snow gall also showed that VFD could protect the color of materials well. Δ*E* represents the color difference between dried licorice and fresh licorice in different ways, the smaller the Δ*E* value, the closer the color of the sample is to that of fresh licorice, which is determined by the L*, a*, and b* values [35]. The results showed that the color of licorice changed significantly after drying in all methods and that significant changes in the b* value contributed most of the change in Δ*E*.

The brightness of the dried samples of licorice depends closely on the drying method. According to our data, VFD has the best color protection effect on licorice tablets, which indicates it can be effectively use to dry high-quality food. This is consistent with the research results Lai Wen et al. [36] on understory ginseng.

### 3.4. Effect of Drying Methods on the Browning Index of Licorice

Browning is an important characteristic of drying quality of licorice. The browning of licorice is mainly caused by the oxidation of total phenols and other active substances and the Maillard reaction [37]. As shown in Table 3, the degree of browning of licorice samples after drying with VFD and VPD were the lowest because the Maillard reaction was prevented in a vacuum environment. In HAD and IR-HAD, the direct contact between licorice and oxygen and their longer drying times led to greater browning, thus, enhancing the occurrence of enzymatic browning. This was consistent with the results from Luntraru et al. [38]. The samples of licorice dried using MVD also had a high degree of browning because the drying speed of MVD was fast and the high power easily caused the licorice to cook, resulting in color degradation, which is consistent with the results from Li Xiaofeng et al. [39], who used MVD to dry *Phyllanthus emblica*.

### 3.5. Effect of Drying Methods on the TPC and TFC of Licorice

Phenols and ketones are important nutrients in licorice [40], but they are prone to thermal oxidation and degradation in the drying process, thus reducing the nutritional quality of licorice.

**Figure 3:**
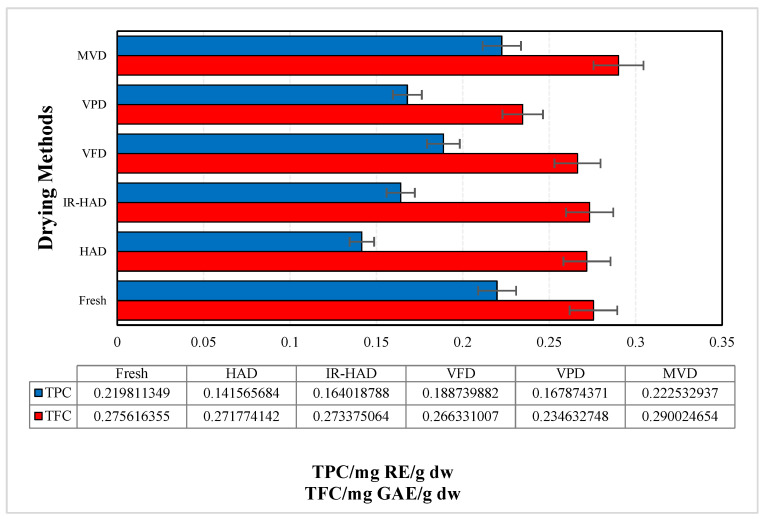
TPC and TFC of licorice at different drying methods.

As shown in Figure 3, our results showed that total flavonoid content (TFC) content in licorice samples after HAD, IR-HAD, or VFD was the same as in fresh licorice samples, there was no apparent damage. The TFC content in licorice samples after MVD drying was the highest compared to other drying method samples and even higher than in fresh licorice samples. This might be due to the very short drying time and short exposure time in a hotter environment, which minimized the destruction of TFC components. In addition, high temperature caused the thermal degradation of phenolic compounds, which promoted the increase of total polyphenol content, and is consistent with the results from Alkaltham et al. [41]. The VPD licorice sample had the lowest TFC content, which may be because it took a long time to dry and the long-term high-temperature environment led to TFC loss.

Unlike the TFC test results, different drying methods resulted in significant differences in TPC content of licorice. Specifically, from highest TPC content to lowest, the drying methods were: MVD, VFD, VPD, IR-HAD, and HAD. In fact, there were no significant changes in flavonoid content after MVD compared to that of fresh licorice. However, VFD retained TPC content in licorice, which might be because the low temperature and vacuum environment could effectively prevent the decomposition of substances and reduce the loss of easily oxidizable components. This was consistent with the results from Wang Di et al. [42]. IR-HAD and VPD licorice samples had the second-highest TPC content, while HAD samples had the lowest. This may be because licorice was exposed to high-temperature air for a long time with sufficient oxygen, which accelerated the oxidation process and led to the reduction of flavonoid content [43]. 

### 3.6. Effect of Drying Methods on the Active Ingredient of Licorice

**Figure 4:**
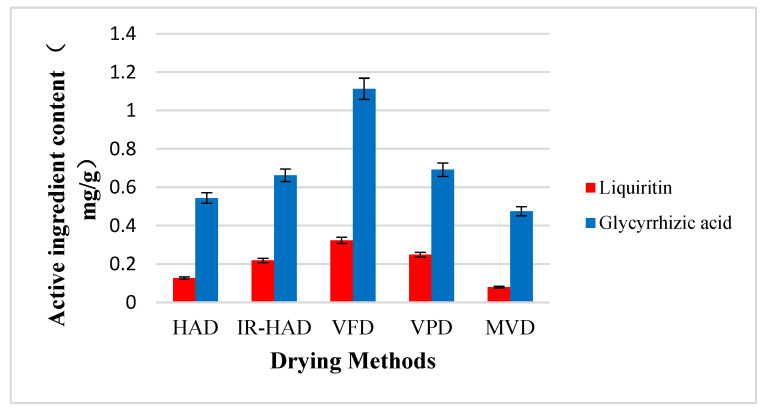
Liquiritin and glycyrrhizic acid of licorice at different drying methods.

As can be seen from the Figure 4, the contents of the main active components of liquiritin and glycyrrhizic acid in the samples of licorice under five different drying methods all changed greatly, and the contents decreased to some extent. VFD resulted in the smallest loss of liquiritin and glycyrrhizic acid in the samples of glycyrrhiza under five different drying methods, followed by IR-HAD and VPD. The HAD samples’ liquiritin content was only 50% of that in VFD samples. In addition, the contents of liquiritin and glycyrrhizic acid were the least in MVD dried samples, and the loss was the greatest. Thus, it can be seen that VFD has a significant protective effect on the main active components of glycyrrhiza, which is consistent with the results from the study by Li Hui et al. [44] on the effects of different drying methods on the content of glycyrrhiza alcohol extract.

## 4. Conclusions

In this study, we assessed the effects of HAD, IR-HAD, VFD, MVD, and VPD on the drying characteristics and quality of licorice. Our findings revealed that the different drying methods had differing effects on the drying characteristics, color, total phenol and total flavones contents, and effective components (liquiritin and glycyrrhizic acid) of licorice. MVD was found to have the shortest drying times and highest effective water diffusion coefficient, and VFD was found to have the longest drying times and lowest effective water diffusion coefficient. Although HAD and IR-HAD were characterized by more rapid drying rates, the quality of the resulting licorice was relatively poor and there were considerable losses of the main effective components of liquiritin and glycyrrhizic acid. Regarding the color of the samples, the best results were obtained using VFD and VPD, which also resulted in the lowest degree of browning. These findings revealed that VFD can contribute to good retention of total phenol, total ketone, and liquiritin and glycyrrhizic acid; however, the cost is high and the process is time-consuming. The highest contents of total phenol and ketone were detected in samples subjected to microwave vacuum drying, even though this method resulted in the lowest contents of liquiritin and glycyrrhizic acid, and the drying process proved difficult to control. In terms of the overall drying characteristics and quality of licorice, VFD was the optimal drying process. However, further studies will be necessary to make this process economically viable (viz. costs, energy consumption, and efficiency) and facilitate mass production.

## Figures and Tables

**Table 1:** Linear equation of content of active components and peak area of licorice after drying.

*Major Active Ingredient*	*Linear Equation*	*R^2^*
Liquiritin	y = 34484 *X* + 1017.8	0.9956
Glycyrrhizic acid	y = 11946 *X* + 223.73	0.9958

Note: y: peak area m^2^; *X*: content mg/mL.

**Table 2:** Effective water diffusivity under different drying methods.

*Drying Method*	*Linear Regression Equation*	*R* ^2^	*D*_eff_ (10^−9^ m^2^/s)
HAD	*lnMR* = −0.02617 *X* − 0.2610	0.9342	23.86
IR-HAD	*lnMR* = −0.02203 *X* + 0.02218	0.9963	20.10
VFD	*lnMR* = −0.01263 *X* − 0.1396	0.9446	11.52
MVD	*lnMR* = −0.1525 *X* + 0.1246	0.9895	139.06
VPD	*lnMR* = −0.01889 *X* − 0.5833	0.8323	17.23

Note: *X*: drying time h.

**Table 3:** The color parameters and browning index of licorice under different drying methods.

*Drying Method*	a*	b*	L*	Δ*E*	*Browning Index* */(Abs/g d.m.)*
HAD 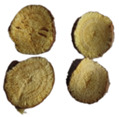	−1.86 ± 0.05 ^c^	19.97 ± 0.15 ^a^	64.54 ± 0.05 ^a^	25.40 ± 0.02 ^a^	0.554 ± 0.01 ^a^
IR-HAD 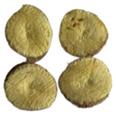	−1.24 ± 0.06 ^d^	24.07 ± 0.07 ^c^	71.57 ± 0.18 ^b^	17.40 ± 0.01 ^c^	0.489 ± 0.01 ^c^
VFD 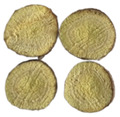	−2.11 ± 0.02 ^b^	25.28 ± 0.02 ^d^	78.10 ± 0.09 ^d^	10.99 ± 0.05 ^d^	0.330 ± 0.01 ^e^
VPD 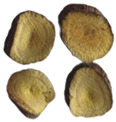	−3.05 ± 0.06 ^a^	23.59 ± 0.07 ^b^	73.21 ± 0.18 ^c^	16.25 ± 0.05 ^c^	0.377 ± 0.01 ^d^
MVD 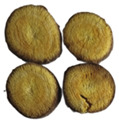	−1.67 ± 0.02 ^cd^	22.87 ± 0.02 ^b^	69.05 ± 0.09 ^b^	20.18 ± 0.03 ^b^	0.522 ± 0.01 ^b^

Note: a*: Red green value. b*: Yellow blue value. L*: Brightness. Δ*E*: color difference.

## Data Availability

Data is contained within the article.
